# Surface nanocrystallization of wood particles from biomass waste for regenerated isotropic wood with excellent properties

**DOI:** 10.1093/nsr/nwab096

**Published:** 2021-05-26

**Authors:** Helmut Cölfen

**Affiliations:** Department of Chemistry, University of Konstanz, Germany

Materials are the cornerstone of human society. Environmentally friendly and sustainable bio-based materials are the key to achieving sustainable development, which is urgently needed to meet global challenges [1–4]. Different from petroleum-based materials, bio-based plant materials are derived from carbon dioxide in the atmosphere, which is fixed by the photosynthesis of plants to form biomaterials like wood. The use of these materials by humans can be traced back to ancient times. However, the existing materials processed from bio-based raw materials are still very limited, mainly including particle board, shaving board, wood-plastic or wood-inorganic materials, etc. [5]. These materials are severely restricted by their dependence on petroleum-based adhesive, complex manufacturing processes and insufficient mechanical performance. How to achieve efficient utilization of bio-based materials and process them into high-performance materials is still a huge challenge.

**Figure 1.**
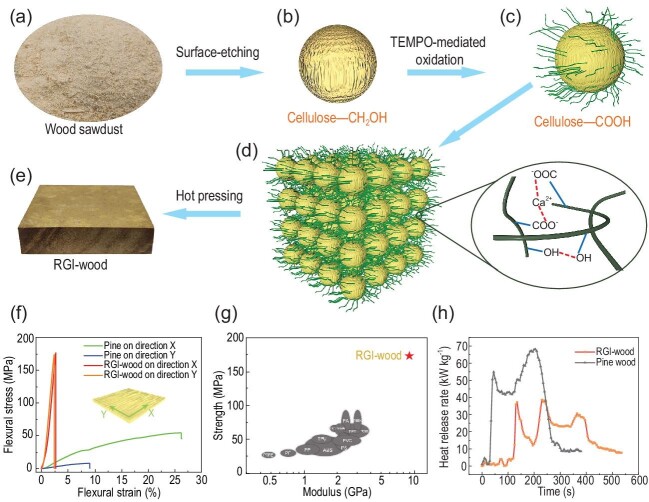
Schematic of the preparation and properties of regenerated isotropic wood. (a) Natural wood particles. (b) Surface-etched wood particle with cellulose microfibers exposed from the surface. (c) Surface nanocrystallized wood particle (SNWP) with numerous cellulose nanofibers expanded from the surface. (d) Assembly of SNWP induced by Ca^2+^ and hydrogen bonds. (e) The obtained RGI-wood by hot-pressing. (f) Flexural stress-strain curves of RGI-wood and natural wood in both directions. (g) Ashby plot: compared to other polymer-based materials, RGI-wood has outstanding mechanical performance. (h) The heat release rate curves of RGI-wood and natural pine wood given by cone calorimetry test showing that RGI-wood releases less heat during combustion. Adapted from Ref. [6].

A team led by Shu-Hong Yu at the University of Science and Technology of China has reported a high-performance sustainable regenerated isotropic wood (RGI-wood), composed of surface nanocrystallized wood particles (SNWP) by an efficient bottom-up strategy with micro/nanoscale structure design [6]. In this work, wood particles with lots of cellulose nanofibers expanded from their surfaces are firstly prepared through a novel surface nanocrystallization method (Fig. 1a–c). This method allows only the cellulose on the surface of microscale wood particles to be transformed into cellulose nanofibers. This micro/nanoscale structure design results in the SNWPs, which can be processed as microparticles but bound to each other like cellulose nanofibers through strong hydrogen bonds and additional electrostatic bonds (Fig. 1d) followed by hot pressing. The strong bonds between SNWPs make RGI-wood a sustainable bio-based material with excellent mechanical properties outperforming wood (Fig. 1e).

RGI-wood has an isotropic high flexural strength (∼170 MPa) and modulus (∼10 GPa), which exceeds the limitation of the anisotropic mechanical properties of natural wood (Fig. 1f). Also, RGI-wood has further performance advantages such as fracture toughness, ultimate compressive strength, hardness, impact resistance, dimensional stability and fire retardancy (Fig. 1h). Because of the strong interaction of SNWPs, RGI-wood needs no extra binder, completely overcoming the risks to the environment and human health caused by resins in particle boards, shaving boards or wood-plastic materials. As an all-natural high-performance sustainable material, RGI-wood is superior to traditional plastics in mechanical properties, which allows RGI-wood to be a strong competitor to petroleum-based plastics in daily life (Fig. 1g). Although the RGI-wood is not as strong as the ‘super wood’ prepared from bulk wood by partial lignin and hemicellulose removal and hot pressing [7], it has the great advantage of being based on waste particles, which allows easy generation of objects of any size and shape. Moreover, SNWP can perform as a great structural binder with a three-dimensional nano-network, thus this versatile bottom-up strategy can be used to construct a series of bulk functional composites. For instance, by mixing SNWPs with carbon nanotubes before pressing, the obtained conductive smart RGI-wood has the potential to be used for electromagnetic shielding or self-heating wallboard for smart buildings.

The surface nanocrystallization method reported by Yu *et al.* is an exciting and universal approach to treat wood biomass waste to create a high-performance sustainable material, which has broad application potential in the fields of green building and home furnishing, etc. It is expected that further similar methods will be developed to treat biomass waste and enrich the large-scale preparation methods of biomass-based high-performance materials towards a more sustainable planet.

***Conflict of interest statement*.** None declared.
